# Impact of the “WeChat Cloud Service” Option for Patients in an Emergent Intensive Care Unit During an Epidemic in Tai Zhou China

**DOI:** 10.3389/fmed.2021.833942

**Published:** 2022-02-03

**Authors:** Jie Wang, Jie Qin, Tao-Hsin Tung, Jianping Chen, Ningyu Zheng, Lili Lu, Yingying Jin

**Affiliations:** ^1^Emergency Department, Taizhou Hospital of Zhejiang Province Affiliated to Wenzhou Medical University, Linhai, China; ^2^Evidence-Based Medicine Center, Taizhou Hospital of Zhejiang Province Affiliated to Wenzhou Medical University, Linhai, China

**Keywords:** pandemic, COVID-19, WeChat, EICU, delirium

## Abstract

To explore the application and effect of “WeChat cloud service” in the emergency intensive care unit (EICU) in the context of an epidemic, we examined 774 patients admitted to an EICU between February 2020 and June 2021. Patients admitted from February 2020 to December 2020 were selected as the control group (*n* = 503) and those from January 2021 to June 2021 comprised the observation group (*n* = 271). There were no statistically significant differences in gender, age, disease, and length of stay in the EICU between the groups. The control group received the general (routine) daily service, such as communicating with families through in-person information transmission, and receiving self-provided drugs and daily supplies during the specified visiting time; the observation group received the “WeChat cloud service” providing the chance of communication, supplies, and payment through the platform at any time. We used a *T*-test and χ2-test to analyse the incidence of delirium, labour costs, and patient and family satisfaction throughout ICU treatment for comparison. Results indicated that the observation group had lower labour costs, less incidence of delirium, and greater patient and family satisfaction than the control group. The “WeChat cloud service” was beneficial for preventing and controlling coronavirus disease 2019 during the epidemic and providing an improved patient experience.

## Introduction

The severe acute respiratory syndrome coronavirus causes an infectious disease known as coronavirus disease 2019 (COVID-19) that mainly involves pulmonary lesions. It is characterised by rapid onset, strong infection, and rapid changes in the physical condition of infected persons (1, 2). As of the time of the writing of this paper, the National Health Committee has incorporated pneumonia caused by COVID-19 as a Class B infectious disease as stipulated in the “Law of the People's Republic of China on Prevention and Control of Infectious Diseases,” and adopted prevention and control measures for Class A infectious diseases. In the period during the COVID-19 pandemic, medical institutions have implemented prevention and control regulations and suggestions by stopping or strictly restricting visits by hospitalised patients' families (Office of the National Health Commission, Office of the State Administration of Traditional Chinese Medicine. Diagnosis and Treatment Protocol for Novel Coronavirus Pneumonia [trial version 6] at http://www.kankyokansen.org/uploads/uploads/files/jsipc/protocol_V6.pdf). Intensive care unit (ICU) patients have reduced immunity and a heightened risk of nosocomial infection, which are the key points of epidemic prevention and control (3). Thus, some nursing modes, such as traditional visits, are no longer suitable for providing nursing care to severe patients, and it is necessary to explore new nursing modes that actively create a good treatment environment for patients and meet the various needs of patients and their families (4, 5). “WeChat cloud service” is the integration of a “cloud visit” wherein the online delivery of hospitalisation expenses, online purchases of daily supplies for patients, and free delivery to the hospital are all managed over the WeChat platform. Therefore, we assessed the use of the WeChat cloud service with patients in an emergency ICU (EICU) to determine if it can offer superior comfort and humanistic care for patients and their families by reducing the incidence of delirium and improving the satisfaction of patients and their families.

## Materials and Methods

### Participants

A total of 774 patients admitted to the EICU of Taizhou Hospital of Enze Medical Centre in Zhejiang province, China, from February 2020 to June 2021 were selected for participation in this study. Inclusion criteria were patients (1) who were hospitalised in the EICU for more than 24 h; (2) who provided informed consent for themselves and their families to participate; (3) who had consciousness or Richmond Agitation Sedation Scale (RASS) > −3; (4) who could read and fill in the questionnaire by themselves; and (5) whose families had smartphones and could skilfully use WeChat functions. Exclusion criteria were patients who (1) had disputes with the EICU or died while in the EICU; (2) did not agree, or whose families did not agree, to participate or cooperate; and (3) dropped out during their time in the EICU. A total of 503 patients admitted from February 2020 to December 2020 were selected as the control group, and 271 patients admitted between January 2021 and June 2021 were selected as the observation group. There were no statistically significant differences in gender, age, disease, and length of stay in the EICU between the two groups (see Table 1). This study was reviewed and approved by the hospital ethics committee.

**Table 1:** Patient demographics and their conditions requiring treatment in the EICU (*n* = 774).

**Characteristics/conditions**		**Control group**	**Observational group**	***x*** ^**2**^ **s/*t***	***P***
Gender	Male	340	190	0.517	0.472s
	Female	163	81		
Age (years)		62.71 ± 17.08	63.36 ± 18.94	−0.482	0.63
Disease or condition	Trauma—non-surgical	142	68	7.637	0.266
	Trauma—surgical	56	23		
	Sepsis	84	47		
	Cardiovascular disease	63	52		
	Cerebrovascular diseases	55	27		
	Poisoning	33	15		
	Other	70	39		
Average length of stay in EICU (days)		7.36 ± 8.10	6.48 ± 5.98	1.703	0.089

### Study Design

In this study, the method of before and after comparison was adopted. The time period was taken as the cut-off point, and the study samples were selected. The conventional nursing mode (control group) and wechat cloud service nursing mode (observational group) were, respectively, adopted. Table 1 showed that there was no statistical difference between observational and control groups.

### Sample Size Determination

The study design were based on the literature reviews and integrated the resources of the hospital. The wechat cloud service system with service items was established. All courses were applied in keeping with the principles of our institutional ethics committee and in accordance with the Declaration of Helsinki. All of the participants' data were kept anonymous. This study was approved by the Institution Review Board of Taizhou Hospital of Zhejiang Province in China.

For the determination of study sample, we exported the data from the EICU electronic nursing shift system. Patients with conscious and RASS score >-3 were screened out by Excel (*n* = 774). According to the patient information, ICDSC delirium score of the patient during EICU hospitalisation was derived from the electronic nursing shift system for statistical analysis of the data. The satisfaction questionnaire is a self-designed questionnaire with a total of 10 mandatory items, and each item is divided into very satisfied, relatively satisfied, satisfied and dissatisfied. The paper and wechat qr code scanning methods are used to investigate, and the final results are statistically analysed with each result accounting for more than 30%, as shown in the figure. Labour cost data: Before and after wechat cloud service, according to doctor-patient communication and business needs during EICU treatment, the average number of patients' family members returning to and from the hospital was counted for statistical analysis.

### Procedure

The control group was offered the general daily service. Under this service, the EICU arranges visits from 15:00 to 15:30 every Monday, Wednesday, Friday, and Sunday when patients' families can visit outside the EICU window if they have negative nucleic acid test results. Before the visit, the nurses made various preparations for each patient, including adjusting their posture; providing basic nursing monitoring, treatment, and support services; checking inpatient account arrears; and managing the quantity of self-provided drugs. Meanwhile, the attendants checked the patient's daily supplies, including nursing pads and disposable gloves. During the WeChat visit, the patient communicates with his or her family through in-person information transmission. Meanwhile, the nurses and attendants communicate with a family member one-on-one according to the situation regarding any inpatient account arrears, the patients' self-provided drugs as verified by doctors, and an account of the daily supplies.

The observation group was offered the “WeChat cloud service” through which patients in the EICU could communicate with their families online during the visiting time specified every day or whenever needed. The procedure was as follows: (1) Preliminary preparation: Applying for the department's mobile phone and WeChat account used for special communication and ensuring that the WeChat accounts of directors, head nurses, responsible nurses, and tube bed doctors, including first-line and second-line doctors of EICU, are added to the department's WeChat account. The head nurse was responsible for establishing a total of 13 WeChat communication groups according to the number of beds using the department's mobile phone and WeChat account. Next, the director, head nurse, responsible nurse, and tube bed doctors joined the WeChat communication groups. The patients' families could join the corresponding communication groups by scanning a QR code. (2) Implementation: (i) After each communication group was established, a group announcement was issued to inform the patients' families about related matters such as not initiating video chatting by themselves. (ii) From 14:00 to 15:00 every day, the designated nurse responsible recorded a video of 2 min or less describing the patient's current condition for each severely ill patient in their charge. Meanwhile, for conscious patients, the designated responsible nurse scheduled video calls according to the needs of each patient. (iii) The EICU arranged visits from 15:00 to 15:30 every Monday, Wednesday, Friday, and Sunday, when the patient's families could visit outside the EICU window upon providing a negative nucleic acid test result. During the visit, video calls were arranged according to the needs of the patient and their families. (iv) In the communication group, the patient's families could ask via text or voice about any concerns they had regarding the patient's treatment and condition outside of the specified times (15:30 to 16:00 on Monday, Wednesday, Friday, and Sunday), and the doctors or nurses could reply later (whenever they were able to). (v) The purchase list of disposable living supplies and payment QR codes was provided according to the needs of patients. Their families could scan the payment QR code directly and pay for disposable living supplies, which were delivered at no cost. (vi) When an inpatient account balance was insufficient, the primary nurse communicated with the family through the communication group to confirm the payment amount and provide the payment QR code to charge. The communication platform also provided online purchases and in-hospital distribution of self-provided drugs. (vii) After the patient was transferred out of the EICU, the responsible nurse sent the patient and their family a QR code to access the satisfaction questionnaire, informed them about how to leave the group, and provided timely updates of the names of active members. (viii) It was uniformly stipulated that the communication group of the patient in the EICU will be called the “communication group of xx in Bed x” and the idle group will be called the “communication group in Bed x.”

### Observation Index

The Intensive Care Delirium Screening Checklist (ICDSC) for patients in the EICU with consciousness or RASS ≥ −3 was used to evaluate their delirium symptoms; a score of ICDSC > 3 indicated the occurrence of delirium. The incidence of delirium was calculated as the number of patients with delirium/the total number of patients included in the evaluation. The incidence of delirium was statistically compared between the two groups.

The satisfaction of patients and their families was measured using a patients' satisfaction questionnaire created by the EICU and included patient's feedback on, nursing quality, medical service attitude, and medical quality. The satisfaction evaluation was completed by the patients and their families using a QR code or fill in the paper questionnaire described in the questionnaire. Next, we exported the evaluation data and tabulated recovery rates and scores: the recovery rate of the satisfaction questionnaire = the number of satisfaction questionnaires collected per month/the total number of satisfaction questionnaires given out (which is equal to the number of transferred patients from the EICU per month).

The number of visits to the hospital patients' families needed was determined according to the condition of the patients, patient needs, and the needs of medical staff. We counted the number of visits family members made to the hospital and calculated the difference between the visits (the control group visits—the observation group visits).

### Statistical Analysis

In this study, all data were analysed using SPSS version 23.0 (IBM Corporation, Armonk, NY, USA). The descriptions of all of the measurement data (patients' age, length of stay in the EICU) and counting data (patients' gender, disease, incidence of delirium, recovery rate of the satisfaction questionnaire) were expressed as mean ± SD and number (%), respectively. For univariate analysis, the two-sample independent *t*-test method and χ^2^-test were adopted to assess differences in the mean value of continuous variables and percentage of categorical variables between observational and control groups. A *p* < 0.05 was considered to represent a statistically significant difference between two test populations.

## Results

### Incidence of Delirium

According to the ICDSC delirium score system, patients with consciousness or RASS ≥ −3 were assessed promptly if the patients experienced consciousness changes. A score of ICDSC > 3 indicated the occurrence of delirium. The statistical results between the two groups are shown in Table 2.

**Table 2:** The incidence of delirium between the control group and “WeChat” group (*n* = 774).

**Item**	***n***	**Cases with delirium *n***	**Incidence of delirium *%***	***x*** ^**2**^ **/*T***	***P***s
Control group	503	89	17.60%	6.665	0.010
Observation group	271	29	10.70%		

### Patient and Family Satisfaction

**Figure 1:**
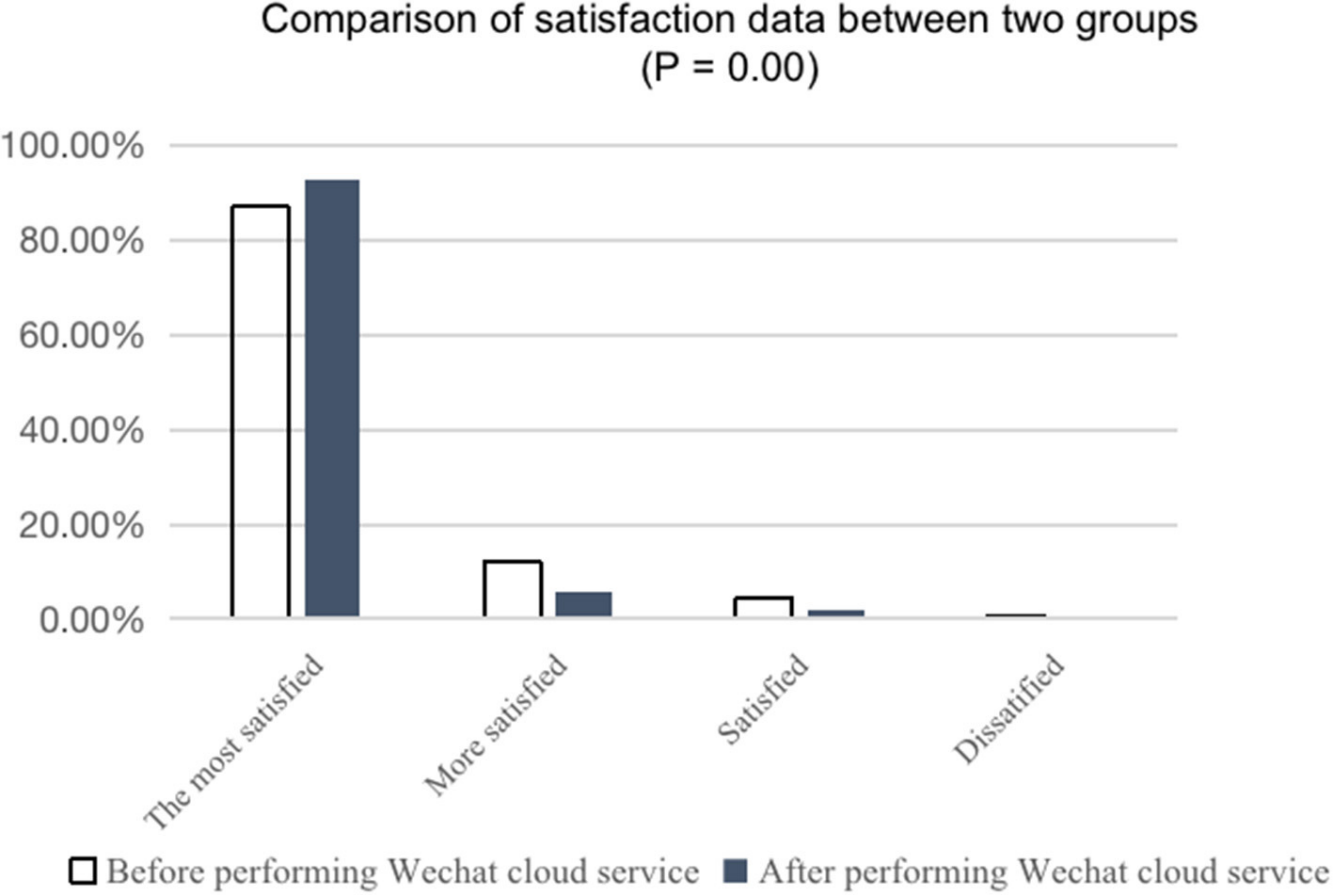
Comparison of “satisfaction” data between the two groups.

The satisfaction evaluation was completed by scanning the QR code or Fill in the paper questionnaire for the self-report satisfaction questionnaire in the WeChat cloud service communication group. Next, the satisfaction data were exported for analysis. The results are as shown in Figure 1.

### Hospital Travel Labour Costs for Patients' Families

After the implementation of the WeChat cloud service, the average number of patients' families needed at the hospital was reduced by 4 ± 2.18 compared with the control group. Thus, the labour cost of hospital travel for families of patients using the WeChat option was extremely low.

## Discussion

Patients in the EICU have critical conditions and high mortality rates (3). To ensure that the patients have adequate rest time and prevent cross-infection, the management mode of “no company, no visitors” is often adopted in these units. However, the establishment of the biological-psychological-social medical mode has highlighted the importance of the social and psychological needs of patients and their families and their impact on biological health (6–8). Hence, visitation is recognised as important for emotional communication between patients and their families in addition to the need for communication between medical staff and patients' families (9). The new epidemic era inevitably brought about the exploration of new visiting modes (10). With the development of communication technology and the popularity of smartphones and the WeChat application, video visitation, including WeChat video visitation, has been explored for use in intensive care units (ICUs) in recent years (11–17). On the basis of various studies on “cloud visits,” we examined a WeChat cloud service platform that encompasses chat and payment functions for patients and their families. The platform was constructed to integrate group-building, video chatting, and payment processing.

Patients in the EICU are also often prone to delirium due to disease and predisposing factors. Delirium aggravates the condition of illness, is not conducive to treatment, and can delay a patient's recovery (18). The prevention and treatment of delirium require a systematic diagnosis and treatment programme composed of multiple projects, objectives, and methods. Currently, ABCDEF and eCASH strategies with delirium emphasise that family care and maximising humanistic care are important (19–21). Some studies (22–25) have found that it can significantly promote the management of delirium by prolonging ICU visiting time and increasing communication opportunities with patients and families. Meanwhile, the consensus among delirium experts is that paying attention to communication with patients and their families reduces the occurrence of delirium and promotes the recovery of delirium (18). Using the cloud visiting function of the WeChat cloud service makes communication with patients and families more flexible and convenient as it is not limited by time and region. In addition, our results indicate it is effective for the prevention and treatment of delirium in EICU patients.

Using the payment function of the WeChat cloud service makes it possible to purchase patients' living supplies and self-provided drugs, and make hospitalisation payments online. Meanwhile, it can reduce the number of visits of patients' families; such a reduction is needed to save labour costs and meet the requirements of epidemic control by reducing gathering among hospital personnel and among members of the general public within the hospital when a family member is admitted. In addition, the recovery rate of the satisfaction questionnaire increased from 41.5 to 81.5% by scanning the QR code instead of the paper questionnaire, and the satisfaction scores of patients and families improved.

### Limitations

There were several limitations should be discussed in this study. Firstly, despite the benefits of the WeChat service reported in this study, only one hospital was included, and it is located in a county-level area where the technological and cultural sophistication of patients and their families is relatively low. Thus, there were certain limitations in the use of WeChat cloud services for some patients, especially older adult patients who are less likely to use smartphones and may need a younger person to assist, thereby creating an indirect means of communication between the older person, the hospital, and the patient or other family members. Secondly, additional instruction or practise may be needed for people with different cultural levels to successfully adopt the technology for use for patients in EICUs. Meanwhile, the “WeChat cloud service” is used to establish communication groups according to the number of beds. The information of patients being admitted into and moving out of the hospital, and the participation and withdrawal of families should be updated timely by a special person to ensure ordering. Thirdly, it is difficult to investigate whether between the experimental group and the control group there were differences in all medical-physiological variables related to the health status of the patient during the period of hospitalisation in the ICU (therefore on the progression of the disease and on healing), that is, to assess whether, between the two groups, there were significant variations in some parameters, detectable with the common equipment present in the ICU, which could have a relationship with the implementation of the “weChat” system namely with this direct and continuous communication with family members. Fourthly, although the satisfaction questionnaire is a self-designed questionnaire, it can be used after reliability and validity test, it was difficult to exclude the error caused by the satisfaction measurement results. At the same time, the questionnaire design does not distinguish between family members and patients themselves, which may also produce certain bias to the results of the survey. Finally, this study only focused on the overall satisfaction of the questionnaire, and there is no sub-dimensions results. Future studies with further verify the effectiveness of wechat cloud service in patients' satisfaction would make these results more convincing.

## Conclusion

The application of the “WeChat cloud service” in EICU can help hospitals meet the psychological needs of patients and their families, reduce the incidence of delirium in patients in EICU, improve the satisfaction among patients and their families, and reduce the cost of labour. Additionally, the WeChat cloud service can effectively reduce gatherings, which is especially critical during an epidemic era. Thus, the service is recommended as worthy for application in clinics.

## Data Availability Statement

The original contributions presented in the study are included in the article/supplementary material, further inquiries can be directed to the corresponding author/s.

## Ethics Statement

The studies involving human participants were reviewed and approved by Medical Ethics Committee of Taizhou Hospital, Taizhou Zhejiang Province. The patients/participants provided their written informed consent to participate in this study.

## Author Contributions

JW conducted the literature search, designed the study, and collected the data and interpreted it. JQ administrated the project. T-HT revised the manuscript. JC, NZ, LL, and YJ interpreted the data and edited critically revised the manuscript. All authors contributed to the article and approved the submitted version.

## Conflict of Interest

The authors declare that the research was conducted in the absence of any commercial or financial relationships that could be construed as a potential conflict of interest.

## Publisher's Note

All claims expressed in this article are solely those of the authors and do not necessarily represent those of their affiliated organizations, or those of the publisher, the editors and the reviewers. Any product that may be evaluated in this article, or claim that may be made by its manufacturer, is not guaranteed or endorsed by the publisher.
